# A Review of the Use of Data Analytics to Address Preeclampsia in Ecuador Between 2020 and 2024

**DOI:** 10.3390/diagnostics15080978

**Published:** 2025-04-11

**Authors:** Franklin Parrales-Bravo, Lorenzo Cevallos-Torres, Leonel Vasquez-Cevallos, Rosangela Caicedo-Quiroz, Roberto Tolozano-Benites, Víctor Gómez-Rodríguez

**Affiliations:** 1 Grupo de Investigación en Inteligencia Artificial, Facultad de Ciencias Matemáticas y Físicas, Universidad de Guayaquil, Guayaquil 090514, Ecuador; lorenzo.cevallost@ug.edu.ec; 2 Centro de Estudios para el Cuidado Integral y la Promoción de la Salud, Universidad Bolivariana del Ecuador, Km 5 ½ vía Durán—Yaguachi, Durán 092405, Ecuador; rtolozano@ube.edu.ec; 3 SIMUEES Simulation Clinic, Universidad Espíritu Santo, Samborondón 092301, Ecuador; leonelvasquez@uees.edu.ec; 4 Instituto Superior Tecnológico Urdesa (ITSU), Av. Pdte. Carlos Julio Arosemena Tola Km 2 ½, Guayaquil 090615, Ecuador; vgomez@itsu.edu.ec

**Keywords:** preeclampsia, clinical decision support systems, disease diagnosis, data analytics, descriptive, diagnostic, predictive, prescriptive

## Abstract

Preeclampsia is one of the leading causes of maternal and perinatal morbidity and mortality worldwide. The incidence of preeclampsia in Ecuador is approximately 51 cases per 1000 pregnancies. Despite advances in medicine, its diagnosis and management remain a challenge due to its multifactorial nature and variability in its clinical presentation. Data analytics offers an innovative approach to address these challenges, allowing for better understanding of the disease and more informed decision-making. This work review examines peer-reviewed studies published during the last decade that employed descriptive, diagnostic, predictive, and prescriptive analytics to evaluate preeclampsia in Ecuador. The review focuses on studies conducted in healthcare institutions across coastal and highland regions, with an inclusion criterion requiring sample sizes greater than 100 patients. Emphasis is placed on the statistical methods used, main findings, and the technological capabilities of the facilities where the analyses were performed. Critical evaluation of methodology limitations and a comparative discussion of findings with global literature on preeclampsia are included. The synthesis of these studies highlights both progress and gaps in predictive analytics for preeclampsia and suggests pathways for future research.

## 1. Introduction

Preeclampsia remains one of the leading causes of maternal and perinatal morbidity and mortality worldwide, currently accounting for 10 to 15% of all maternal deaths worldwide [1,2]. In Ecuador, the burden of this syndrome is compounded by various geographical, socioeconomic and technological factors [3]. The country’s unique territorial characteristics, including its diverse altitudes—ranging from coastal lowlands to Andean highlands—present distinct challenges in the diagnosis and management of preeclampsia. For instance, women residing in high-altitude regions, such as the Andean highlands, are at increased risk of developing preeclampsia due to hypoxia and reduced oxygen availability, which can exacerbate hypertensive disorders during pregnancy [4]. In addition, socioeconomic disparities, limited access to healthcare in rural areas, and cultural factors further complicate the management of preeclampsia in Ecuador [4,5]. As in other situations, we can use information and communication technologies to improve the situation [6,7,8,9]. However, significant gaps remain, particularly in the integration of these technologies into clinical practice in rural and high-altitude regions [10].

Early Ecuadorian research focused on understanding epidemiological patterns [11,12,13], risk factors [14,15,16,17], and clinical outcomes of preeclampsia [18,19], particularly in high-altitude regions and among vulnerable populations such as adolescents and indigenous communities. For example, studies conducted before 2020 emphasized the role of altitude in exacerbating hypertensive disorders during pregnancy, and women living in the Andean highlands experienced higher rates of preeclampsia compared to those of coastal regions [20,21,22]. Furthermore, early research highlighted the importance of prenatal care and nutritional interventions, such as folic acid and calcium supplementation [23,24,25], in reducing the risk of preeclampsia.

A recent study adopted a holistic application of all analytics approaches (descriptive, diagnostic, predictive, and prescriptive) to study the number of preeclampsia inpatient beds in a hospital in Guayaquil [26]. In fact, in recent years, an increasing trend has been observed in the integration of these analytical methodologies into clinical practice [27,28,29,30]. However, to the best of our knowledge, there is no study that reviews the current status of the use of data analytical approaches in the field of preeclampsia and in the Ecuadorian context.

This review aims to synthesize the findings of studies published between 2020 and 2024 that have applied any data analytics approach to improve understanding, diagnosis, prevention, and treatment of preeclampsia in Ecuador. The primary objective of this review of the literature is to synthesize current evidence on the application and effectiveness of various analytical methods in predicting and managing preeclampsia within Ecuador. Through a multidisciplinary approach, we discuss case studies, data analysis techniques, and technological tools that can transform clinical care and reduce complications associated with this condition.

This review is relevant because it highlights both the strengths and limitations of analytical approaches, describing topics where further development and improved data quality are needed. The following sections describe the methodology used to select the literature, summarize the findings of the studies, discuss the strengths and limitations of data analysis approaches, and provide conclusions that recommend future research and practice.

## 2. Related Work

### 2.1. Data Analytics Approaches

According to [31,32], analytics may be classified as descriptive, diagnostic, predictive, and prescriptive (DDPP). What happened? Why did that occur? What is going to occur? Each of these analytics attempts to answer the questions, and what can be done to make it happen? The authors assert that descriptive and diagnostic analysis has the lowest level of uncertainty because most of the parts are known. Prescriptive and predictive analytics, on the other hand, have higher degrees of uncertainty as they seek to provide insightful analysis of the future. Moreover, prescriptive analytics suggests a proactive decision, whereas descriptive, diagnostic, and predictive analytics provide information for decision support systems [33].

**Figure 1:**
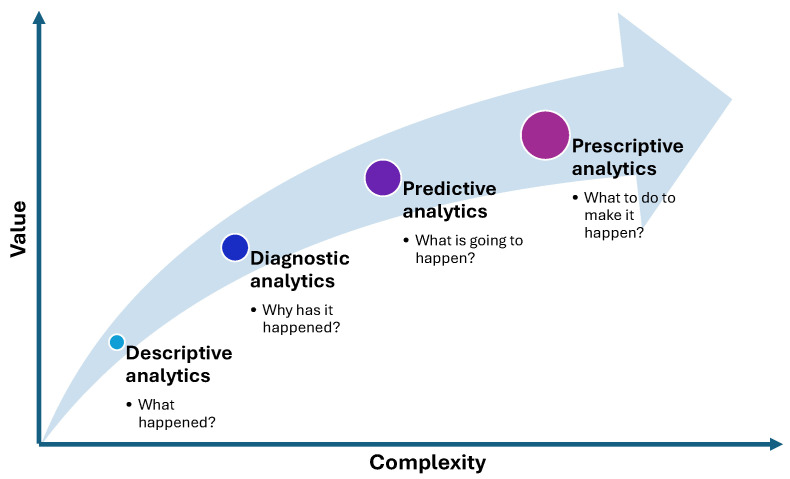
Data analytics approaches, value, and complexity.

Inspired by the results shown in [31,32], Figure 1 summarizes the aforementioned classification and lists the questions that each form of analytics raises.

Descriptive analytics focuses on summarizing historical data to understand past trends and patterns. Diagnostic analytics dives deeper to identify the causes behind these trends. Predictive analytics uses statistical models and machine learning to forecast future outcomes based on historical data. Finally, prescriptive analytics provides actionable recommendations by leveraging optimization and simulation techniques. Together, these methods enable organizations to make data-driven decisions, optimize processes, and enhance overall business performance.

### 2.2. Related Literature Reviews

In the field of preeclampsia, several review works have been carried out in the last 5 years. However, to our knowledge, there is no work that reviews work that has made use of any of the DDPP approaches to improve the diagnosis and/or management of the disease.

In Table 1, we will present a summary of relevant review works carried out within the Ecuadorian context. We will also consider those Latin American studies that have included Ecuador in their analysis.

The main findings of Table 1 can be summarized in the following points:

- Adolescent pregnancies in Ecuador are associated with a high prevalence of complications, including hypertensive disorders, anemia, and low birth weight.
- Expectant care for severe preeclampsia remote from term may result in better neonatal outcomes, such as higher birth weight and lower rates of neonatal death and intensive care unit (ICU) admission.
- The prevalence of preeclampsia in Latin America and the Caribbean is estimated at 6.6%, with substantial heterogeneity across studies and regions.
- A higher frequency of preeclampsia and gestational hypertension is observed at higher altitudes, with complications such as lower birth weight, HELLP syndrome, and ICU hospitalization.
- Aspirin and calcium/vitamin D supplements are effective preventive measures for preeclampsia.
- Magnesium sulfate remains the most effective treatment for managing preeclampsia in Ecuador.

All in all, the main findings from Table 1 underscore the significant burden of preeclampsia in Ecuador, particularly among high-risk groups such as adolescents and women living at high altitudes. While effective treatments like magnesium sulfate and preventive measures like aspirin and calcium supplementation are available, there is a need for further research, improved healthcare access, and targeted interventions to improve the management of preeclampsia in Ecuador.

A limitation of the reviews presented in Table 1 is that, although it is true that most of them address reviews of articles with descriptive or diagnostic approaches, it is not possible to know in detail which analytical techniques or approaches are being used within Ecuador, or which are being used less, nor can the strengths and weaknesses of each approach be known within the study of Ecuadorian preeclampsia.

### 2.3. Motivation

According to [26], the integration of advanced analytics and machine learning models can offer promising avenues to improve the prediction, prevention, and management of preeclampsia in the country. Therefore, this work will address a systematic review of the studies on patients with preeclampsia conducted over the last 5 years, grouping them by the different analytical approaches (DDPP). In this way, we can know what of DDPP analytics are being widely used and what approaches need to be strengthened to improve the management and prevention of preeclampsia in Ecuador.

Specifically, the review addresses the following research questions (RQs):

- From the studies grouped by each analytical approach…
  − RQ1: What are the techniques used?
  − RQ2: What are the key findings?
  − RQ3: What are the public health implications?
- RQ4: Are the findings in line with what the world literature says?
- RQ5: What are the strengths and limitations of each analytical approach?

## 3. Methodology

This literature review was conducted using a systematic approach to identify peer-reviewed journal articles published within the past five years (2020–2024) that focus on preeclampsia analytics in Ecuador. The search strategy was designed to isolate studies that employed descriptive, diagnostic, predictive, and prescriptive analytics techniques in the evaluation of preeclampsia, with specific attention paid to sample sizes greater than 100 patients. The selected studies also include research performed in both public and private health institutions covering diverse regions: coastal and highlands.

It is important to mention that the final report followed the PRISMA-ScR (Preferred Reporting Items for Systematic reviews and Meta-Analyses extension for Scoping Reviews) guidelines [40]. The protocol was pre-registered on the Open Science Framework (OSF) portal.

### 3.1. Databases and Search Strategy

Multiple electronic databases were searched, including PubMed, Scopus, and regional repositories specific to Latin America such as Scielo, Redalyc, LILACS, and “Biblioteca virtual en salud Ecuador” (BVS-Ecuador). Table 2 presents their company names and addresses (city, country) of the databases and software used.

Keywords utilized in the search comprised “preeclampsia”, “Ecuador”, “descriptive”, “diagnostic”, “predictive”, and “prescriptive”. Boolean operators and various truncation strategies were implemented to broaden the search to encompass robust studies on analytics methodologies. The search was conducted on 17 February 2025. Table 3 presents the number of articles retrieved using each search strategy applied.

### 3.2. Inclusion Criteria

For the present study, we selected articles that met the following criteria:

- Studies published within the last five years.
- Research conducted in Ecuador in both public and private healthcare institutions.
- Studies involving sample sizes of over 100 patients.
- Articles published in indexed, peer-reviewed scientific journals.
- Papers that report on the use of descriptive, diagnostic, predictive, or prescriptive analytics in the context of preeclampsia.

### 3.3. Exclusion Criteria

In this study, we discarded the articles that contained any of the following points:

- Studies with insufficient methodological detail for evaluation.
- Review articles or articles that do not directly address research on a hospital population.
- Research exclusive to unintended datasets (e.g., simulated data or case reports with sample sizes fewer than 100 patients).
- Articles not written in English or Spanish.

### 3.4. Data Extraction and Synthesis

**Figure 2:**
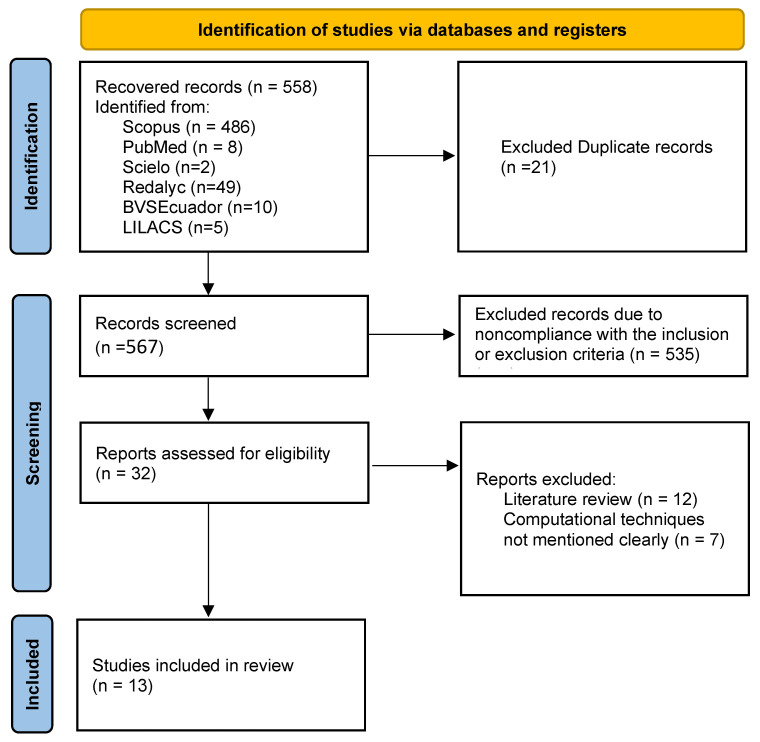
Flowchart of study selection according to the PRISMA guidelines.

Figure 2 presents the flowchart of study selection according to the PRISMA guidelines [40]. The primary stages of data extraction included reviewing article abstracts, full-text assessments, and subsequent categorization by analytics type used. The extracted data items encompassed bibliographic details (title, authors, type of document, publication year), study objectives, population, sample size, applied techniques or methods, maternal and fetal results, and any noted methodological limitations. Data synthesis involved tabulating this information and conducting a narrative analysis to compare outcomes across different analytical approaches.

## 4. Findings

The literature search yielded a total of 558 potential articles; following a detailed screening process (as shown in Figure 2), 13 studies met the inclusion criteria. The following sections summarize the key findings, public health implications, and conclusions, grouping the works by each analytic approach employed.

### 4.1. Descriptive Analytics

Descriptive analytics can be a fundamental tool to understand the current status of preeclampsia in Ecuador, helping to explore epidemiological data, prevalence, risk factors, clinical outcomes, and trends associated with this condition, with the aim of providing a solid basis for public health decision-making.

#### 4.1.1. Selected Descriptive Studies

Table 4 presents the studies that have met the inclusion criteria defined for this manuscript.

#### 4.1.2. Techniques (RQ1)

**Figure 3:**
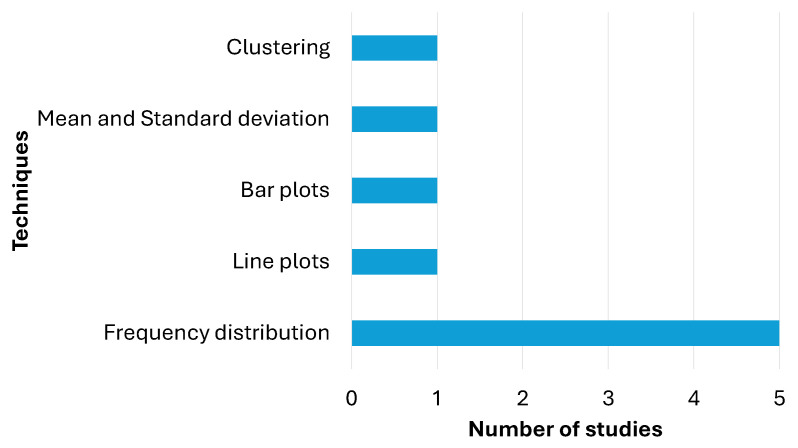
Descriptive techniques.

Descriptive techniques are essential tools for summarizing and visualizing data effectively. From Table 4, we can see that frequency distribution is the most commonly used technique, with a total of five applications, highlighting its relevance for organizing and understanding the occurrence of values within a dataset. Other techniques, such as line graphs and bar graphs, have been used by one study, being useful for visualizing trends over time and comparing categories, respectively. Furthermore, the combination of mean and standard deviation is used in one study, allowing the analysis of both the central tendency and the dispersion of the data. Finally, clustering is also used on one occasion, demonstrating its usefulness for grouping data based on similarities. Figure 3 presents a summary of the findings described here.

#### 4.1.3. Key Findings (RQ2)

Below, we will mention the key findings provided by studies presented in Table 4.

- Incidence and Severity:
  − The incidence of preeclampsia in Ecuador is approximately 51 cases per 1000 pregnancies [4].
  − A consistent upward trend in Ecuadorian preeclampsia cases has been observed in the last years [4,42].
  − Preeclampsia is more widespread across both low- and high-altitude areas, while eclampsia is predominantly found at lower altitudes [4].
- Complications:
  − Complications were most prevalent during the puerperium, followed by labor and delivery, and pregnancy [41].
  − Maternal complications include HELLP syndrome, renal failure, liver failure, and cerebral edema [4].
  − Fetal complications include intrauterine growth restriction, preterm birth, and perinatal death [4,41].
- Geographical and Ethnic Variations:
  − Preeclampsia is more prevalent in certain ethnic groups, such as Montubios and Afro-Ecuadorians, particularly at higher altitudes [4].
  − The disease’s impact is more severe in rural and underserved areas, where access to healthcare [4,41] and education [44] is limited.

#### 4.1.4. Public Health Implications (RQ3)

Among the articles reviewed, the following actions are suggested to be taken into account:

- Early detection during prenatal visits, especially around 32 weeks, is crucial for managing preeclampsia [4].
- Improved access to healthcare, better diagnostic tools, and targeted interventions for high-risk groups are essential to reduce the burden of preeclampsia and eclampsia in Ecuador [4].

#### 4.1.5. Conclusion

Preeclampsia remains a critical challenge in Ecuador, particularly in high-altitude regions and among specific ethnic groups. Addressing this issue requires a multifaceted approach, including improved healthcare access, early detection, and targeted interventions for high-risk populations. Further research is needed to better understand the underlying causes and to develop effective prevention and management strategies.

### 4.2. Diagnostic Analytics

Diagnostic analytics approaches focused on identifying risk factors and correlating clinical parameters with preeclampsia outcomes. Researchers used regression models, clustering techniques, and correlation analysis to investigate relationships between patient variables and disease severity.

#### 4.2.1. Selected Diagnostic Studies

Table 5 presents the studies that have met the inclusion criteria defined for this manuscript.

#### 4.2.2. Techniques (RQ1)

**Figure 4:**
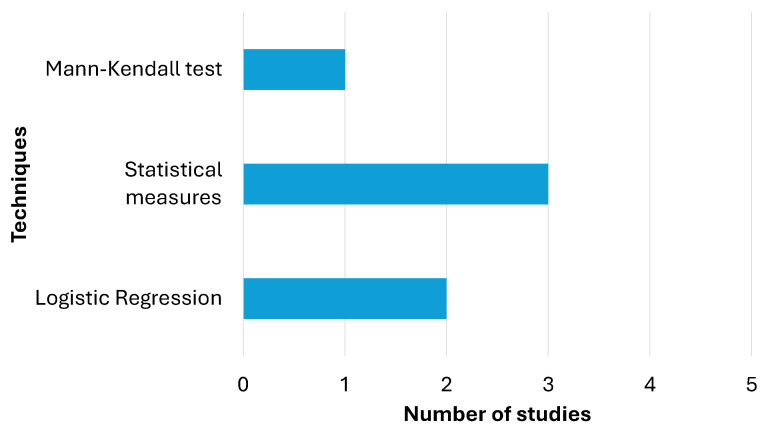
Diagnostic techniques.

Diagnostic techniques are key tools for analyzing and evaluating data to identify significant patterns, trends, or relationships. From Table 5, statistical measures (such as hazard ratio, odds ratio, chi-square, confidence interval, and *p*-value) are the most frequent, with a total of 3 applications, highlighting their importance in summarizing and quantifying key features of data. Logistic regression appears twice, indicating its use in modeling relationships between variables and predicting binary outcomes. Finally, the Mann–Kendall test is used once, demonstrating its application in detecting time trends in data series. Figure 4 presents a summary of the findings described here.

#### 4.2.3. Key Findings (RQ2)

Below, we will mention the key findings provided by studies presented in Table 5.

- Risk Factors:
  − Ethnicity and Altitude: Montubio women living at middle or high altitudes have the highest risk of preeclampsia. Afro-Ecuadorians also show an increased risk associated with altitude [4].
  − Micronutrient Intake: Iron and folic acid consumption during pregnancy is protective [5].
  − Maternal Age: Both younger (10–14 years) and older (≥35 years) maternal ages were associated with an increased risk of preeclampsia [4]. First pregnancy and low schooling are significant risk factors in adolescents [45].
  − Geographical Distance: Women living more than 20 km from healthcare facilities have a higher risk of developing both preeclampsia and eclampsia [4].
  − Family History: A family history of preeclampsia increases the risk [45].
  − Obesity and Overweight: Higher BMI (≥25) is associated with an increased risk of preeclampsia [45].
  − Prenatal care: Adequate prenatal care (more than five visits starting in the first trimester) significantly reduces the risk of preeclampsia [5].
  − Socioeconomic disparities: Women with lower income, and lower educational attainment are more susceptible to pregnancy complications, including preeclampsia [5,26].
  − Seasonal Variation: Preeclampsia cases tend to increase during the rainy (January to April) and summer (August to November) months in Guayaquil, Ecuador [26].

#### 4.2.4. Public Health Implications (RQ3)

Among the articles reviewed, the following actions are suggested to be taken into account:

- Improve Access to Prenatal Care: Ensure that all pregnant women, especially those in rural and low-income areas, have access to regular prenatal visits starting in the first trimester [4,5].
- Nutritional Support: Provide micronutrient supplementation (e.g., iron and folic acid) and education on healthy diets during pregnancy [5].
- Education and Awareness: Implement educational programs to raise awareness about the importance of prenatal care and early detection of risk factors for preeclampsia [5,45].
- Adolescent Health Programs: Develop targeted health programs for adolescent girls to reduce early pregnancies and improve access to prenatal care [45].
- Weight Management: Promote healthy weight management and nutrition programs for adolescents and pregnant women to reduce the risk of obesity-related complications [45].
- Altitude-Specific Interventions: Develop targeted interventions for pregnant women and neonates in high-altitude regions, such as oxygen conditioning and specialized prenatal care [4,10].
- Reduce Geographical Barriers: Improve access to obstetric care, especially in rural and remote areas, to reduce the risk of preeclampsia and eclampsia [4].
- Community Health Programs: Strengthen community health programs to provide education and support for pregnant women, especially in high-risk areas [4].
- Seasonal Variation: Seasonal awareness campaigns and targeted interventions during high-risk periods can help mitigate the impact. For example, increased monitoring and resource allocation during these months could reduce complications.

#### 4.2.5. Conclusion

Statistical techniques in diagnostic analytics mainly involved multivariate logistic regression to extract the most significant parameters.

The findings highlight the multifactorial nature of preeclampsia, with risk factors ranging from socioeconomic and demographic factors to biological and environmental influences.

Despite careful fitting of the models, selected studies noted potential limitations due to incomplete clinical records and regional variations in data collection practices.

### 4.3. Predictive Analytics

These studies aimed to create models that could forecast the likelihood of preeclampsia occurrence based on patient profiles and environmental factors. For instance, Gomez et al. (2019) utilized decision trees and random forests to predict adverse maternal and fetal outcomes in women with preeclampsia. Predictive models were developed using training and validation data segmented by geographical region and healthcare institution type.

#### 4.3.1. Selected Predictive Studies

Table 6 presents the studies that have met the inclusion criteria defined for this manuscript.

#### 4.3.2. Techniques (RQ1)

**Figure 5:**
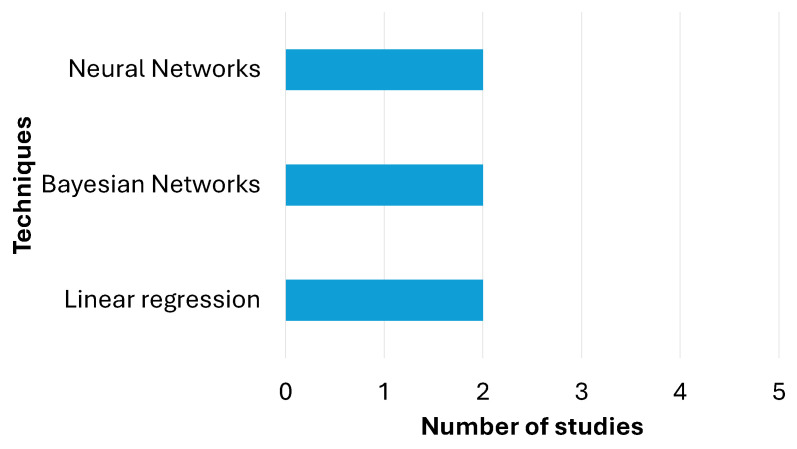
Predictive techniques.

Predictive techniques are fundamental tools for anticipating future outcomes based on historical data or identified patterns. In Table 6, a balanced use of three main methods can be observed: linear regression, Bayesian networks and neural networks, each with a frequency of two applications. Linear regression is used to predict linear relationships between variables, while Bayesian networks allow for modeling uncertainties and probabilistic dependencies to make predictions. On the other hand, neural networks stand out for their ability to handle complex and non-linear problems through machine learning. This distribution reflects a diverse and complementary approach, combining traditional statistical methods, probabilistic models and advanced artificial intelligence techniques for prediction. Figure 5 presents a summary of the findings described here.

### 4.4. Key Findings (RQ2)

Below, we will mention the key findings provided by studies presented in Table 5.

- Homocysteine as a Predictor: Homocysteine levels were not found to be a reliable predictor of preeclampsia in the first study, possibly due to widespread folic acid supplementation [48].
- Predictive Algorithms: The predictive algorithm from the Hospital Clinic of Barcelona demonstrated high accuracy in identifying preeclampsia risk, even without angiogenic biomarkers, making it suitable for low-resource settings [46].
- Machine Learning Models: Bayesian network classifiers, particularly the TANcl algorithm, showed high accuracy in predicting preeclampsia risk, with key risk factors including advanced maternal age, hypertension, and lifestyle factors [1].
- Clinical Implications: Both predictive algorithms and machine learning models can be valuable tools for early detection of preeclampsia, especially in resource-limited settings, but further validation and integration of biomarkers may improve accuracy [1,46,48].

These findings highlight the potential of machine learning models in improving the prediction, management, and prevention of preeclampsia, ultimately contributing to better maternal health outcomes.

#### Public Health Implications (RQ3)

Among the articles reviewed, the following actions are suggested to be taken into account:

- Resource Management: Predictive models can help hospitals manage inpatient beds and emergency room arrivals more effectively, especially during peak demand periods [26,47].
- Early Intervention: Identifying high-risk patients early (e.g., those with hypertension, tobacco use, and diabetes family history) allows for timely preventive measures, reducing the incidence of preeclampsia and improving maternal and perinatal outcomes [1,46].

### 4.5. Prescriptive Analytics

Prescriptive analytics studies explored how analytical approaches could be used to inform treatment protocols and clinical decision-making processes. Research in this area incorporated simulation models, optimization algorithms, and decision analysis frameworks to recommend interventions aimed at mitigating risks where preeclampsia was predicted.

#### 4.5.1. Selected Prescriptive Studies

Table 7 presents the studies that have met the inclusion criteria defined for this manuscript.

#### 4.5.2. Techniques (RQ1)

Within preeclampsia, prescriptive analytics techniques can be considered for the following:

- Mathematical Optimization: Mathematical models are used to find the best possible solution within a set of constraints. For example, in the context of hospital beds, it can be used to determine how many beds to allocate to each department to maximize efficiency and minimize waiting times [26].
- Simulation: Virtual models can be created that mimic the behavior of a real system to test different scenarios and decisions. For example, the temporary closure of a hospital unit for preeclampsia can be simulated and its impact on patient care and referral capacity measured [26].
- Rule-Based Decision Models: Predefined rules (e.g., using fuzzy logic) can be defined to recommend specific actions based on particular conditions. For example, if the number of occupied beds exceeds 90% of capacity, refer patients to other hospitals or activate emergency protocols [49].

The only article of Table 7 has made use of Bayesian structural time-series models to quantify a scenario, namely, the effects of such a closure on the number of inpatient beds needed for preeclampsia care. With it, the manuscript provides actionable recommendations for hospital management, focusing on improving resource allocation, optimizing clinical processes, and preparing for potential emergencies. These insights are crucial for ensuring timely and effective care for preeclampsia patients, particularly in resource-constrained settings.

Despite promising findings, the integration of prescriptive models into clinical workflows was challenged by technological constraints, particularly in rural highland facilities.

#### 4.5.3. Key Findings (RQ2)

Below, we will mention the key findings provided by the unique work presented in Table 7.

- Given the projected increase in preeclampsia hospitalizations, hospitals should allocate more inpatient beds during peak months.
- Early risk identification and intervention could lower the need for hospitalization.
- The study simulated the impact of closing the hospitalization unit in 2024, similar to the COVID-19 scenario. The closure would result in an average of 130.33 monthly referrals, highlighting the need for alternative strategies to manage bed occupancy during crises.
- The study suggests a possible link between preeclampsia incidence and respiratory/infectious diseases, warranting further research.
- The study highlights the importance of big data analytics in healthcare management.

#### 4.5.4. Public Health Implications (RQ3)

Among the unique article reviewed, the following actions are suggested to be taken into account:

- Enhancing Resource Allocation: Decision-makers should use predictive analytics to anticipate demand and optimize hospital capacity.
- Strengthening Non-Hospital Care Services: To reduce hospital burden, outpatient monitoring and community-based management programs should be expanded.
- Developing Emergency Response Plans: Future pandemics or disasters should not completely disrupt preeclampsia care. Policies should ensure that maternity services remain operational even during health emergencies.
- Addressing Potential Risk Factors: Public health campaigns should promote nutritional awareness, prenatal care, and lifestyle changes to mitigate risk factors such as obesity.
- Utilizing Data-Driven Decision Making: Integration of machine learning models into hospital management systems can improve patient outcomes and optimize resource use.

#### 4.5.5. Conclusion

In the last 5 years, little work has been conducted on the prescriptive approach to hospital management of preeclampsia in Ecuador. The unique research presented by [26] underscores the growing burden of preeclampsia and the need for data-driven hospital management. Using predictive analytics to forecast hospital bed demand and prescriptive analytics to optimize healthcare policies can help mitigate maternal mortality risks associated with preeclampsia.

All in all, prescriptive analytics can transform preeclampsia management by turning data insights into actionable strategies for improving patient care and resource utilization. By predicting future trends and recommending optimal interventions, hospitals and policy-makers can reduce maternal deaths, enhance early diagnosis, and optimize healthcare delivery for pregnant women at risk of preeclampsia.

## 5. Discussion

The synthesis of the studies reviewed highlights several overarching themes regarding the use of analytics in preeclampsia research in Ecuador. Each analytics methodology offers unique insights and benefits, yet they also carry distinct limitations that affect their clinical applicability. This section compares outcomes with global literature on preeclampsia and discusses the strengths and weaknesses of each approach.

### 5.1. Comparison with Global Literature on Preeclampsia (RQ4)

When compared to the global literature, key similarities and differences emerge. The prevalence of preeclampsia varies worldwide but generally affects 2–8% of pregnancies [1,50]. In Ecuador, a notably higher incidence of 51 cases per 1000 pregnancies (5.1%) is present, which aligns more with high-risk populations in low- to middle-income countries (LMICs).

Moreover, established risk factors such as maternal age, obesity, family history, and comorbidities like hypertension and diabetes are consistent with findings from studies in the US [50,51,52], Europe [53], Asia [54], and Africa [55]. Furthermore, the role of high altitude as a contributing factor is particularly relevant to Ecuador and has also been studied in Andean and Himalayan populations [37,56,57,58], where oxygen deprivation may contribute to hypertensive disorders during pregnancy.

In addition, Ecuadorian studies emphasize geographic and ethnic disparities in Ecuador, which are also observed globally. Studies in Africa [59,60] and South Asia [61,62,63] similarly highlight socioeconomic and healthcare access limitations as major contributors to maternal health disparities. The disproportionate burden of preeclampsia in rural and indigenous communities aligns with research in Peru [64], India [65], and sub-Saharan Africa [66], where healthcare infrastructure remains a challenge [67].

All in all, the comparison with global literature on preeclampsia reveals both similarities (risk factors) and unique aspects (prevalence of preeclampsia) of the Ecuadorian context. These findings underscore the importance of addressing both universal and localized risk factors to reduce the burden of preeclampsia, particularly in underserved populations.

### 5.2. Evaluating the Strengths and Limitations of Each Analytics Approach (RQ5)

Descriptive analytics provides a foundational understanding of preeclampsia epidemiology, including prevalence, risk factors, and geographical distribution. The use of visualization techniques (e.g., histograms, GIS mapping) helps in identifying patterns and trends. However, it is limited to summarizing historical data and cannot provide causal insights or predictive capabilities. The reliance on retrospective data may introduce biases, and the lack of real-time data limits its utility in dynamic healthcare settings [68].

Diagnostic analytics helps identify root causes and risk factors for preeclampsia, such as ethnicity, altitude, and socioeconomic status. Techniques like regression analysis and hypothesis testing provide valuable insights into the relationships between variables. However, this kind of analysis relies heavily on the quality and completeness of data [69]. In Ecuador, incomplete clinical records and regional variations in data collection practices may limit the accuracy of diagnostic models. Additionally, diagnostic analytics does not offer predictive or prescriptive capabilities, not providing proactive information [70].

Predictive analytics, particularly machine learning models, shows promise in identifying women at high risk of preeclampsia early in pregnancy. Models like Bayesian networks and decision trees have demonstrated high accuracy in predicting preeclampsia risk, enabling early intervention. However, predictive models require large, high-quality datasets for training and validation [71]. In Ecuador, the lack of standardized EMRs and real-time data integration poses challenges. Additionally, predictive models may not generalize well across different populations or regions without external validation [72].

Prescriptive analytics offers actionable recommendations for resource allocation, crisis management, and preventive measures. For example, prescriptive analytics have been considered in [26] to optimize bed management during peak demand periods and to develop targeted interventions during high-risk seasons. However, the integration of prescriptive analytics into clinical workflows is hindered by technological constraints [73], particularly in rural and highland regions. While some urban hospitals have digital records, many rural facilities still rely on paper-based documentation [74], limiting data integration and real-time analytics [75]. All in all, the limited availability of real-time data and advanced computational infrastructure limits the immediate applicability of prescriptive methods.

To conclude, the strengths and limitations of each analytics approach underscore the importance of technological investments and standardized data collection practices. Future research should focus on enhancing data quality, validating predictive models, and integrating analytics into clinical workflows to improve maternal and perinatal outcomes.

## 6. Conclusions

This literature review has synthesized evidence from peer-reviewed studies conducted in Ecuador over the past five years that applied descriptive, diagnostic, predictive, and prescriptive analytics in the context of preeclampsia. The analysis indicates that while each analytics approach offers distinct contributions to understanding and managing preeclampsia, their effectiveness is significantly influenced by institutional technological capabilities and regional differences.

Descriptive and diagnostic analytics have provided an essential foundation, elucidating baseline epidemiological factors and correlations with adverse maternal and fetal outcomes. Predictive analytics, leveraging modern machine learning algorithms, has shown considerable promise in forecasting preeclampsia with high accuracy. Prescriptive analytics bridges prediction and intervention by offering tailored management recommendations; however, its utility is curtailed by challenges in real-time data integration and technological infrastructure.

Limitations of the reviewed studies—such as the reliance on retrospective data, variations in methodology, and technological discrepancies—must be acknowledged and addressed in future investigations. Enhancing the quality and consistency of data collection, coupled with a greater emphasis on integrating analytics into clinical workflows, will likely result in even greater improvements in maternal and fetal outcomes. In summary, while the analytics approaches reviewed here are promising, a concerted effort is required to close the technological and methodological gaps that currently hinder their optimal application in Ecuadorian healthcare settings.

## 7. Implications for Future Research and Clinical Practice

The incorporation of advanced analytics in preeclampsia research offers a promising avenue for early detection and improved management of this high-risk condition. Future research should address the following areas:

- Enhanced Data Collection: Establishing standardized EMR systems across all institutions can mitigate data variability and improve the reliability of descriptive and predictive analytics. Past studies, such as those by [1,4,76], have highlighted the challenges of incomplete clinical records and regional variations in data collection. Based on these findings, future research should focus on creating unified data collection protocols that ensure consistency in urban and rural healthcare facilities.
- Advanced Model Validation: Future studies should incorporate rigorous external validation frameworks to ensure that predictive models are generalizable between different populations and regions. For example, in [46], the high accuracy of predictive models in urban settings is demonstrated, but their applicability in rural areas remains untested. Drawing from global studies, such as [72], which emphasize the importance of external validation, future research should aim to validate models in diverse Ecuadorian populations, including high-altitude and indigenous communities.
- Integrative Analytics Approaches: Combining descriptive, diagnostic, and predictive models can lead to more robust prescriptive analytics that inform practical treatment protocols. Past studies, such as [26], have shown the potential of integrating multiple analytics approaches to optimize hospital resource management. Future research should build on these findings by developing integrated decision support systems that leverage real-time data to provide actionable recommendations for clinicians.
- Technological Investments: Policy-makers and healthcare administrators must invest in modern diagnostic and computational infrastructure, particularly in under-resourced highland regions, to fully leverage the benefits of advanced analytics. Studies like [74] have documented the challenges of transitioning from paper-based to digital records in low-resource settings. Future research should explore cost-effective technological solutions that can be implemented in rural areas, ensuring that all regions of Ecuador benefit from advancements in data analytics.
- Community-Based Interventions: Past research, such as [44], has emphasized the importance of community health programs in reducing the burden of preeclampsia, particularly in underserved areas. Future studies should focus on developing and evaluating community-based interventions that combine education, nutritional support, and early screening to reduce the incidence of preeclampsia in high-risk populations.
- Longitudinal Studies: Although cross-sectional studies have provided valuable information on the risk factors and prevalence of preeclampsia, longitudinal studies are needed to understand the long-term impacts of the condition on maternal and child health. For example, the authors of [53] conducted a retrospective cohort study that highlighted the long-term effects of gestational hypertension on offspring. Similar studies in Ecuador could provide critical insight into the lifelong consequences of preeclampsia and inform targeted interventions.

For clinicians, adopting integrated decision support systems based on validated predictive and prescriptive models can bridge the gap between theoretical analytics and patient care. The translation of these research findings into clinical practice could ultimately reduce the incidence and severity of preeclampsia, leading to improved maternal care and neonatal health outcomes.

## Figures and Tables

**Table 1:** Relevant literature reviews conducted in the last 5 years.

Authors	Year	Countries of Study	Goal	Main Findings	Limitations
Barrera et al. [34]	2024	Ecuador	Analyze new studies on the management and care of pregnant women with preeclampsia to improve prognoses and treatment efficiency.	Magnesium sulfate remains the most effective treatment. Aspirin and calcium/vitamin D supplements are effective preventive measures. Early screening and intervention are recommended.	Limited sample size; reliance on secondary sources; need for further clinical trials to validate findings.
Tite et al. [35]	2024	Ecuador	Identify maternal–fetal complications associated with preeclampsia.	Women aged 20–34 years are at higher risk. Early detection is essential, especially in low-resource settings. Aspirin and calcium are suggested for high-risk women.	Limited to open-access articles, potential selection bias. Only 10 studies reviewed in the last 5 years.
Blanco et al. [36]	2022	Argentina, Bolivia, Brazil, Chile, Colombia, Dominican Republic, Ecuador, Guatemala, Guadeloupe, Haiti, Honduras, Mexico, Nicaragua, Panama, Paraguay, Peru, Puerto Rico, Trinidad and Tobago, Uruguay, Venezuela	To estimate the point prevalence of pregnancy-induced hypertension, preeclampsia, gestational diabetes, low birth weight, and preterm delivery in Latin America and the Caribbean, and evaluate the heterogeneity of the estimates.	Incidences: preeclampsia: 6.6%, gestational diabetes: 8.5%, low birth weight: 8.5% preterm birth: 10.0%, substantial heterogeneity observed in prevalence estimates across studies and by study design.	Limited to 20 out of 43 countries in the region, potentially underestimating heterogeneity. Focused on meta-analyses using all available studies regardless of bias level, which may affect the reliability of estimates. Lack of universally accepted diagnostic criteria for gestational diabetes limits cross-regional comparisons.
Román-Lazarte [37]	2024	Bolivia, Ecuador, Perú, Turquía	To synthesize the evidence on the clinical manifestations and complications of preeclampsia in pregnant women residing at high and very high altitudes.	A higher frequency of preeclampsia and gestational hypertension is observed at higher altitudes. Complications such as lower birth weight, higher frequency of HELLP syndrome and ICU hospitalization are reported.	The review includes studies with low quality of evidence. Specific questions were not asked, which led to mixed results. Databases from some regions such as China were not included, which may have excluded relevant information.
Ledesma et al. [38]	2023	Ecuador	To examine the presence and recurrence of obstetric and perinatal complications in pregnant adolescents in Ecuador, and propose components of a comprehensive prevention program to reduce maternal and perinatal complications.	High prevalence of complications such as anemia, abortion, hypertensive disorders, postpartum hemorrhages, and low birth weight. Need for comprehensive prevention programs including education, emotional support, and access to sexual education and contraceptive methods. Persistent challenges in maternal and neonatal health despite existing prevention programs.	Focused on meta-analyses using all available studies regardless of bias level, which may affect the reliability of estimates. Lack of universally accepted diagnostic criteria for gestational diabetes limits cross-regional comparisons.
Quintero-Ortiz [39]	2021	Egypt, South Africa, United States, Colombia, England, Iraq, Japan, Turkey, Panama, Guatemala, Peru, Mexico, Ecuador, Venezuela	To compare the effects of expectant versus interventionist care in the management of pregnant women with severe preeclampsia remote from term.	Expectant care may result in lower incidence of Apgar scores < 7 at 5 min and higher average birth weight. Expectant care may decrease rates of neonatal death, hyaline membrane disease, and admission to the NICU. No significant differences in maternal mortality, eclampsia, HELLP syndrome, placental abruption, pulmonary edema, or renal failure. Expectant care extended pregnancy by an average of 1 week.	Low quality of evidence due to the nature of included studies. Imprecision in some outcomes. Limited number of studies included, making publication bias assessment unfeasible. High risk of bias in non-randomized studies (NRSs).

**Table 2:** Details of scientific databases considered in this review.

Database	Company/Organization	Address	Software
PubMed	NCBI/NLM (USA)	8600 Rockville Pike, Bethesda, MD, USA	https://pubmed.ncbi.nlm.nih.gov/advanced/ (accessed on 20 February 2025)
Scopus	Elsevier	Radarweg 29, Amsterdam, Netherlands (HQ); 230 Park Ave, New York, NY, USA	https://scopus.com/ (accessed on 20 February 2025)
SciELO	FAPESP/SciELO Network	Rua Dr. Diogo de Faria 1087, São Paulo, SP, Brazil	https://search.scielo.org/advanced/?lang=es (accessed on 20 February 2025)
Redalyc	UAEMex	Instituto de Ciencias Agropecuarias y Rurales, Toluca, Estado de México, Mexico	https://www.redalyc.org/home.oa (accessed on 20 February 2025)
LILACS	BIREME/PAHO/WHO	Rua Botucatu 862, São Paulo, SP, Brazil	https://lilacs.bvsalud.org/es/ (accessed on 20 February 2025)
BVS-Ecuador	BIREME/PAHO + Ecuadorian institutions	Varies (typically Quito, Ecuador)	https://bvs-ecuador.bvsalud.org/ (accessed on 20 February 2025)

**Table 3:** Search strategy to extract Ecuadorian preeclampsia studies published since 2020.

Analytic Approach	Search Strategy	Database	Results
Descriptive	(“preeclampsia” OR “pre-eclampsia”) AND (“Ecuador” OR “Ecuadorian”) AND (“descriptive” OR “incidence” OR “frequency” OR “prevalence”)	Scopus	210
		PubMed	3
		Scielo	2
		Redalyc	24
		BVS-Ecuador	7
		LILACS	4
Diagnostic	(“preeclampsia” OR “pre-eclampsia”) AND (“Ecuador” OR “Ecuadorian”) AND (“diagnostic” OR “regression” OR “causal”)	Scopus	142
		PubMed	3
		Scielo	0
		Redalyc	18
		BVS-Ecuador	1
		LILACS	0
Predictive	(“preeclampsia” OR “pre-eclampsia”) AND (“Ecuador” OR “Ecuadorian”) AND (“predictive” OR “prediction” OR “classification” OR “classifying”)	Scopus	130
		PubMed	2
		Scielo	0
		Redalyc	7
		BVS-Ecuador	2
		LILACS	1
Prescriptive	(“preeclampsia” OR “pre-eclampsia”) AND (“Ecuador” OR “Ecuadorian”) AND “prescriptive”	Scopus	4
		PubMed	0
		Scielo	0
		Redalyc	0
		BVS-Ecuador	0
		LILACS	0
		Total	558

**Table 4:** Selected descriptive studies.

Authors/Year	Goal	*n*	Data	Technique	Contribution
Tejera et al.(2021) [4]	To study ethnic, geographical, and altitude differences in the incidence of preeclampsia and eclampsia in Ecuador.	15,390	A population-based retrospective study using data from the Ecuadorian National Institute of Statistics and Census (INEC) and the Ministry of Health (2015–2017)	Frequency distribution in absolute and relative values	This study highlights the importance of ethnicity, altitude, and geographical access to healthcare in the prevalence of preeclampsia and eclampsia in Ecuador. It provides valuable insights for targeted public health interventions.
Viteri-Hinojosa and Espinosa-Yépez (2024) [41]	To analyze morbidity due to anesthetic complications in obstetric patients in Ecuador (2018 to 2022)	304	A cross-sectional descriptive study using data from the National Institute of Statistics and Census (INEC) hospital discharge records (2018–2022).	Line plots, bar plots, mean	This study underscores the need for continuous monitoring and training in obstetric anesthesia to reduce complications. It also highlights the impact of external factors, such as the pandemic, on maternal health outcomes.
Parrales et al. (2024) [42]	To characterize preeclampsia patients using descriptive and clustering analysis to aid in the distribution of clinical care and prevention policies.	6406	Electronic medical records (EMRs) from the IESS Los Ceibos Hospital, collected from May 2017 to December 2023.	Frequency distribution in absolute and relative values, clustering	The findings highlight the importance of age and diagnostic profiles in understanding the prevalence and severity of preeclampsia.
Vargas et al. (2021) [43]	To determine the risk factors of hypertensive pregnancy disorders in pregnant women who attended prenatal care at the Universitary Hospital of the City of Guayaquil	104	Pregnant patients who attended the prenatal check-up at the Universitary Hospital of Guayaquil-Ecuador, from January 2019 to January 2020.	Frequency distribution in absolute and relative values	This study highlights that personal and family history of hypertensive disorders was a relevant risk factor, as well as nutrition, first parity, and change of partner.
Hernández et al. (2021) [44]	To determine the risk factors for preeclampsia in pregnant women from communities attended at the Hospital General Puyo.	150	direct observation, review of medical records of 150 pregnant women and survey applied during the period from January 2021 to April 2021.	Frequency distribution in absolute and relative values	This study highlights that the main factor influencing the risk of preeclampsia was the lack of knowledge on the part of the pregnant women due to their low level of education.

**Table 5:** Selected diagnostic studies.

Authors/Year	Goal	*n*	Data	Technique	Contribution
Tejera et al. (2021) [4]	To study ethnic, geographical, and altitude differences in the incidence of preeclampsia and eclampsia in Ecuador.	15,390	A population-based retrospective study using data from the Ecuadorian National Institute of Statistics and Census (INEC) and the Ministry of Health (2015–2017)	Logistic regression and geospatial analysis were used to assess risk factors and spatial clusters of preeclampsia and eclampsia	This study highlights the importance of ethnicity, altitude, and geographical access to healthcare in the prevalence of preeclampsia and eclampsia in Ecuador. It provides valuable insights for targeted public health interventions.
Cho et al. (2021) [45]	To examine the prevalence and associated factors of preeclampsia, eclampsia, and gestational hypertension in adolescent women in the period 2019–2020 at the Homero Castanier Crespo Hospital in Azogues, 2021	298	Retrospective data of adolescent women (aged 11 to 19 years) in the period 2019–2020 at the Homero Castanier Crespo Hospital in Azogues.	Association between preeclampsia and risk factors was determined with statistical measures such as odds ratio, chi-square, 95% confidence interval, and *p*-value less than 0.05.	This work highlights that the prevalence of hypertensive disorders in adolescents is high. Its causes may be low level of education, primiparous mothers, family history of preeclampsia, overweight and obesity.
Tite et al. (2024) [5]	To investigate the relationship between prenatal care and the incidence of preeclampsia among pregnant women in Ecuador.	20,648	The study uses data from the National Health and Nutrition Survey (ENSANUT) 2018, which includes a nationally representative sample of 20,648 mothers.	Use of binary logistic regression and multicollinearity tests.	The study finds that adequate prenatal care (more than five visits starting in the first trimester) significantly reduces the risk of preeclampsia. It also highlights that women from rural areas, with lower income, and with lower educational attainment are more susceptible to preeclampsia.
Dueñas et al. (2021) [10]	To investigates the relationship between altitude and neonatal survival among at-risk neonates in Ecuador.	3016	Data from the Ecuadorian Ministry of Public Health’s Surveillance System of Neonatal Mortality, including neonatal deaths registered between January 2014 and September 2017 across 126 public and private healthcare facilities.	The study used Cox proportional hazards models to estimate hazard ratios (HRs) for different altitude strata, adjusting for individual variables (e.g., birth weight, gestational age, Apgar score) and contextual variables (e.g., type of healthcare facility, level of care).	The study highlights the importance of considering altitude as a critical factor in neonatal health, particularly in high-altitude regions. The findings suggest that healthcare interventions and policies should take into account the challenges posed by high altitudes, especially for at-risk neonates.
Parrales et al. (2024b) [26]	To analyze the monthly occupancy of inpatient beds for preeclampsia care at the IESS Los Ceibos Hospital in Guayaquil, Ecuador, from May 2017 to December 2023	6406	Electronic medical records (EMRs) from the IESS Los Ceibos Hospital, collected from May 2017 to December 2023.	Mann–Kendall test to see any consistent upward trend in preeclampsia care.	The study highlights seasonal fluctuations. It also presents anomalies in the increasing trend in the number of preeclampsia care cases due to COVID-19.

**Table 6:** Selected predictive studies.

Authors/Year	Goal	*n*	Data	Technique	Contribution
Guerrero, K. and Abadie, P. (2024) [46]	To evaluate the operational capacity of the Hospital Clinic de Barcelona predictive model for the detection of preeclampsia (PE) risk in a cohort of women treated at IESS Los Ceibos Hospital in Guayaquil, Ecuador.	304	The study used 304 pregnant women at IESS Los Ceibos Hospital in Guayaquil, Ecuador, between August 2018 and August 2019.	Linear regression models from the Hospital Clinic de Barcelona were used for the detection of early and late risk of preeclampsia	The study suggests stratified screening with biomarkers in high-risk subgroups to optimize accuracy without universal screening, adapting resources to local needs in resource-limited settings like Ecuador. The model showed high predictive capacity with an AUC of 0.924, sensitivity of 88.46%, and specificity of 91.37%.
Parrales et al. (2024b) [26]	To analyze the monthly occupancy of inpatient beds for preeclampsia care at the IESS Los Ceibos Hospital in Guayaquil, Ecuador, from May 2017 to December 2023	6406	Electronic medical records (EMRs) from the IESS Los Ceibos Hospital, collected from May 2017 to December 2023.	Techniques such as Dynamic Bayesian Networks (DBNs), Multilayer Perceptron (MLP), and Long Short-Term Memory (LSTM) were used for forecasting the inpatient bed occupancy.	The study highlights the need for better hospital resource management, especially during high-demand periods, and suggests strategies like clinical process redesign and capacity enhancement to reduce inpatient bed demand. The MLP model achieved the lowest MAPE (11.58%) and predicted peaks of 274 and 258 inpatient beds in September and November 2024, respectively.
Parrales et al. (2024c) [47]	To predict emergency room arrivals for preeclampsia at the IESS Hospital del Día Sur Valdivia.	2926	The data included 2926 EMRs on gynecological emergency care carried out between 2019 and 2023.	Techniques such as Extreme Learning Machine (ELM), Multilayer Perceptron (MLP), and Dynamic Bayesian Networks (DBN) were employed.	The study emphasizes the importance of predictive analytics in managing emergency care for preeclampsia, allowing hospitals to allocate resources more effectively and improve patient outcomes. The MLP model achieved the lowest MAPE (17.21%), with an RMSE of 4.21 and MAE of 2.74. It predicted 11 to 13 emergency arrivals due to preeclampsia for September to November 2024.
Parrales et al. (2024d) [1]	To predict and explain the risk of suffering preeclampsia at the IESS Los Ceibos Hospital.	6406	Electronic medical records (EMRs) from the IESS Los Ceibos Hospital, collected from May 2017 to December 2023.	Bayesian network classifiers (BNCs) including Naïve Bayes (NB), Tree-Augmented Naïve Bayes (TANcl), and Semi Naïve Bayes (FSSJ) were used.	The study suggests that early identification of high-risk patients (e.g., those with hypertension, tobacco use, and diabetes family history) can lead to timely interventions, reducing the incidence of preeclampsia and improving maternal health outcomes. The TANcl model achieved the highest accuracy (close to 90%) and was effective in predicting preeclampsia risk. The F1 score and specificity were also high, indicating good performance in identifying both positive and negative cases.
Vargas et al. (2023) [48]	To evaluate the role of serum homocysteine as a predictor of preeclampsia in pregnant women between 12 to 20 weeks of gestation.	312	Patients treated in the outpatient service of the Gynecology and Obstetrics service of the University Hospital of Guayaquil, in Guayaquil-Ecuador. The study period was from 1 October 2018 to 30 October 2019.	Inferential statistics were used for comparative analysis between groups. Categorical data were formed and chi-square was used to establish the association or difference.	The study contributes to the ongoing debate about the role of homocysteine in predicting preeclampsia. Although the results did not support homocysteine as a predictor, the findings are valuable for guiding future research and clinical practice, particularly in low-resource settings where preeclampsia is a significant public health issue.

**Table 7:** Selected prescriptive studies.

Authors/Year	Goal	*n*	Data	Technique	Contribution
Parrales et al. (2024b) [26]	To quantify the effects of a closure on the number of inpatient beds needed for preeclampsia care during 2024 at the IESS Los Ceibos Hospital in Guayaquil, Ecuador	6406	Electronic medical records (EMRs) from the IESS Los Ceibos Hospital, collected from May 2017 to December 2023.	Bayesian structural time-series models.	The study simulated the impact of closing the hospitalization unit in 2024, similar to the COVID-19 scenario. The closure would result in an average of 130.33 monthly referrals, highlighting the need for alternative strategies to manage bed occupancy during crises.
